# Olfactory impairment and probable REM sleep behavior disorder in Parkinson’s disease: clinical relevance to the single- and dual-hit hypotheses

**DOI:** 10.3389/fnagi.2026.1778012

**Published:** 2026-09-22

**Authors:** Chia-Yen Lin, Ting-Hsin Chang, Ling-Yu Lin, Ming-Hong Chang

**Affiliations:** 1 Department of Neurology, Neurological Institute, Taichung Veterans General Hospital, Taichung, Taiwan; 2 Department of Psychiatry, MacKay Memorial Hospital, Taipei, Taiwan; 3 Department of Post-Baccalaureate Medicine and Brain and Neuroscience Research Center, College of Medicine, National Chung Hsing University, Taichung, Taiwan

**Keywords:** anosmia, body-first subtype, brain-first subtype, olfactory impairment, probable REM sleep behavior disorder (pRBD), REM sleep behavior disorder (RBD), single-hit hypothesis

## Abstract

**Background:**

Rapid eye movement (REM) sleep behavior disorder (RBD) and olfactory impairment are common prodromal features of Parkinson’s disease (PD), but the implications of their coexistence remain uncertain. The dual-hit hypothesis proposes concomitant olfactory and enteric involvement, whereas the revised single-hit framework proposes predominantly brain-first or body-first routes. We examined whether probable RBD (pRBD)/anosmia-defined subgroups exhibit distinct clinical profiles.

**Methods:**

In this cross-sectional study, 201 individuals with PD underwent olfactory, cognitive, motor, non-motor, and quality-of-life assessments. Anosmia was defined as a University of Pennsylvania Smell Identification Test score <19 and pRBD as an RBD Screening Questionnaire score ≥6 supplemented by participant and informant interviews. Multivariable models evaluated the main and interactive effects of anosmia and pRBD.

**Results:**

Of 201 participants, 95 (47.3%) were pRBD-positive (pRBD+), 135 (67.2%) had anosmia, and 68 (33.8%) had both. Anosmia prevalence did not differ between the pRBD+ and pRBD-negative (pRBD−) groups (71.6% vs. 63.2%; *p* = 0.208). After adjustment for age, sex, and disease duration, pR+A+ and pR+A− (subgroups defined by probable RBD status [pR+/pR−] and anosmia status [A+/A−]) did not differ in any assessed outcome. The anosmia-by-pRBD interaction was negative for MDS-UPDRS part III (β = −8.3, 95% CI −15.2 to −1.4; *p* = 0.019). Within the pRBD− subgroup, pR−A+ participants had lower MoCA scores than pR−A− participants (β = −2.6; 95% CI −4.1 to −1.0; *p* = 0.001), and the anosmia-by-pRBD interaction was positive for MoCA (β = 2.3, 95% CI 0.0 to 4.5; *p* = 0.049). Anosmia was associated with cognitive impairment in pRBD− participants (OR 2.53, 95% CI 1.00–6.37; *p* = 0.049), but not in pRBD+ participants (OR 1.65, 95% CI 0.60–4.50; *p* = 0.331).

**Conclusion:**

In pRBD+ participants, anosmia was not associated with greater motor severity; in pRBD− participants, it was instead linked to cognitive impairment. Concurrent assessment of olfaction and pRBD may improve clinical phenotyping, but longitudinal validation is required.

## Introduction

Parkinson’s disease (PD) is a heterogeneous neurodegenerative disorder with diverse motor and non-motor phenotypes. Several non-motor features, including rapid eye movement (REM) sleep behavior disorder (RBD), olfactory impairment, depression, and anxiety, may precede the clinical diagnosis of PD (Ishihara and Brayne, 2006; Doty, 2012; Barone and Henchcliffe, 2018).

Hyposmia and anosmia denote different degrees of olfactory impairment. Throughout this study, our prespecified operational definition of anosmia is used (University of Pennsylvania Smell Identification Test [UPSIT] <19). In reference to previous studies, we use the broader term olfactory impairment, retaining the original report’s terminology where the hyposmia–anosmia distinction is relevant. Olfactory impairment is common in PD and has been reported in 60%–90% of diagnosed individuals (Tissingh et al., 2001; Swallow et al., 2016; Berg et al., 2021). However, olfactory performance may not correspond directly to olfactory bulb pathology (Tremblay et al., 2022), because odor identification also engages higher-order olfactory cortex and memory and may reflect more widespread central Lewy pathology (Borghammer et al., 2022). Neuropathological studies have identified an amygdala-predominant pattern in a subset of early Lewy body cases, with α-synuclein pathology centered on the amygdala and transentorhinal cortex and often involving the olfactory bulb (Borghammer and Van Den Berge, 2019; Borghammer et al., 2021, 2022). In aged rats, intragastric seeding of α-synuclein produced pathology in multiple brain regions, including the olfactory bulb (Van Den Berge et al., 2021). A review of non-motor PD subtypes similarly proposed both an olfactory-limbic route and a combined brainstem-plus-olfactory route (Sauerbier et al., 2016).

Conceptual models of PD pathogenesis have evolved from the dual-hit hypothesis to brain-first/body-first subtyping and, more recently, a revised single-hit framework (Braak et al., 2003; Hawkes et al., 2007; Jellinger, 2009, 2012; Horsager et al., 2020; Borghammer et al., 2022). The dual-hit hypothesis proposes independent or concomitant propagation of α-synuclein through olfactory and enteric routes (Hawkes et al., 2007). By contrast, the brain-first/body-first model proposes that pathology begins either in the central nervous system and subsequently spreads to the peripheral autonomic nervous system or follows the reverse sequence (Borghammer and Van Den Berge, 2019; Horsager et al., 2020; Borghammer et al., 2021; Horsager and Borghammer, 2024). In clinical and imaging studies, the body-first subtype is commonly operationalized by premotor RBD confirmed with video-polysomnography (Horsager et al., 2020). The revised single-hit hypothesis further proposes that initial α-synuclein pathology arises predominantly through either a brainstem/body-first route or an amygdala/brain-first route, rather than both routes simultaneously (Borghammer et al., 2022).

Although the body-first subtype is hypothesized to carry a greater central Lewy body burden and therefore more severe olfactory impairment, some studies observed no differences in olfactory function between RBD+ and RBD− PD (Romenets et al., 2012; Sixel-Döring et al., 2014) or between probable RBD-positive (pRBD+) and probable RBD-negative (pRBD−) PD (Liu et al., 2017). Whether anosmia identifies a more severe clinical phenotype across the pRBD+ and pRBD− subtypes also remains unclear. We therefore examined pRBD and anosmia concurrently to determine whether four pRBD/anosmia-defined subgroups differ in motor, cognitive, non-motor, and quality-of-life outcomes and whether the observed profiles are more consistent with a single-hit or dual-hit model.

## Materials and methods

We conducted a cross-sectional study of 201 individuals with PD recruited from the outpatient clinic of Taichung Veterans General Hospital between March 2019 and January 2024. The study was approved by the Institutional Review Board and Ethics Committee of Taichung Veterans General Hospital (IRB certificate no. CE24111C), and all participants provided written informed consent in accordance with the Declaration of Helsinki. The primary inclusion criterion was fulfillment of the Movement Disorder Society clinical diagnostic criteria for idiopathic PD (Postuma et al., 2015). The exclusion criteria were organized into two domains according to their rationale. First, to ensure diagnostic certainty for idiopathic PD, we excluded individuals with (1) conditions associated with secondary or atypical parkinsonism, including dementia preceding parkinsonism, stroke, hydrocephalus, or hypoxic encephalopathy; (2) exposure to antidopaminergic or dopamine-depleting agents at doses and durations consistent with drug-induced parkinsonism; or (3) an unsatisfactory response to levodopa during clinical follow-up. Second, to preserve the validity of the olfactory assessment, we excluded individuals with (4) nasal or sinus disease or (5) antidepressant use, which may alter olfactory function.

### Clinical assessments

#### RBD screening questionnaire (RBDSQ)

Video-polysomnography is the diagnostic gold standard for RBD. In this study, probable RBD (pRBD) was identified using the RBDSQ, as in the Oxford Discovery Cohort Study (Liu et al., 2021). The RBDSQ is a validated 13-item yes/no questionnaire that assesses dream content, dream-enactment behaviors, their consequences, and the presence of neurological disease. A score ≥6 defined pRBD (pRBD+) and <6 defined pRBD−, consistent with the Oxford Discovery Cohort cutoff. Questionnaire responses were supplemented by interviews with participants and, when available, bed partners or family members regarding dream-enactment behavior.

#### Olfactory function

Olfactory function was assessed using the validated traditional Chinese version of the University of Pennsylvania Smell Identification Test (UPSIT) (Doty et al., 1984; Jiang et al., 2014). The test comprises 40 odorants embedded in scratch-and-sniff labels. After releasing each odorant with a pencil tip, participants selected the correct odor from four response options. Scores range from 0 to 40, with lower scores indicating poorer olfactory function. A score < 19 was used to define anosmia. The use of UPSIT for olfactory assessment in this cohort has been described previously (Fang et al., 2023; Lin et al., 2025).

#### Cognition

Global cognition was assessed with the MoCA and MMSE. The MMSE is relatively insensitive to the mild executive and visuospatial deficits commonly observed in PD and is susceptible to ceiling effects, whereas the MoCA is better suited to detecting mild cognitive impairment in this population (Hoops et al., 2009; Dalrymple-Alford et al., 2010). Accordingly, the MoCA was the primary cognitive measure, and cognitive impairment was defined as a score < 26 (Nasreddine et al., 2005; Dalrymple-Alford et al., 2010). The MMSE was analyzed in parallel using the same non-parametric approach, with impairment defined as a score < 26 in accordance with the Movement Disorder Society Task Force Level I criteria for PD dementia (Dubois et al., 2007). Because of its ceiling effect, MMSE results are reported in the Supplementary Material section.

#### Non-motor symptoms scale (NMSS)

Non-motor symptoms were assessed using the 30-item Non-Motor Symptoms Scale (NMSS) (Chaudhuri et al., 2007).

#### PD severity and quality of life

PD-related symptoms were assessed using the MDS-UPDRS (Goetz et al., 2008), which comprises part I (non-motor experiences of daily living), part II (motor experiences of daily living), part III (motor examination), and part IV (motor complications). Quality of life was assessed using the validated Chinese version of the PDQ-39 SI (Peto et al., 1995; Ma et al., 2005).

#### Dopamine transporter imaging

^99m^Tc-TRODAT-1 single-photon emission computed tomography (SPECT) was performed as part of the routine diagnostic work-up, and interpretable reports were available for 191 of the 201 participants. Scans were interpreted clinically by nuclear medicine physicians using a semi-quantitative visual classification of striatal uptake (Huang et al., 2004), which grades caudate and putamen uptake on each side from normal to severe. Inter-rater agreement was not obtained, and quantitative striatal binding ratios were not available. Imaging supported the diagnosis of nigrostriatal degeneration and was analyzed only as a secondary, exploratory comparison.

#### Statistical analysis

Statistical analyses were performed using MedCalc Statistical Software version 23.1.3 (MedCalc Software Ltd, Ostend, Belgium), IBM SPSS Statistics version 21, and RStudio version 2023.06.1+524 for Windows with the PMCMRplus package. Distributional assumptions were assessed using the Kolmogorov–Smirnov test. Continuous variables are summarized as medians and interquartile ranges (IQRs). Normally distributed variables were compared across groups using analysis of variance; other continuous variables were compared using the Kruskal–Wallis test. When the omnibus Kruskal–Wallis test was significant, pairwise comparisons were performed using Conover’s test with Bonferroni adjustment. Categorical variables were compared using chi-squared tests. Participants were cross-classified by pRBD and anosmia status into four subgroups: pR+A+ (pRBD-positive with anosmia), pR+A− (pRBD-positive without anosmia), pR−A+ (pRBD-negative with anosmia), and pR−A− (pRBD-negative without anosmia). Adjusted comparisons among the four subgroups were estimated using generalized linear models that included age, sex, and disease duration as covariates. For each continuous outcome, factorial linear regression models estimated the independent main effects of anosmia and pRBD and their interaction, with adjustment for the same covariates. Cognitive impairment (MoCA < 26) was evaluated using multivariable logistic regression in the full cohort and in models stratified by pRBD status. Factorial linear regression models were adjusted for age, sex, and disease duration, whereas the multivariable logistic regression models were additionally adjusted for levodopa-equivalent daily dose (LEDD). Missing data were not imputed, and all analyses used the available observations for each instrument. All tests were two-sided, and *p* < 0.05 was considered statistically significant.

## Results

Among the 201 participants, 95 (47.3%) were pRBD+, 135 (67.2%) had anosmia, and 68 (33.8%) had both pRBD and anosmia (Table 1). For the purposes of this study, pRBD+ participants were considered more likely to correspond to the body-first subtype and pRBD− participants to the brain-first subtype. Anosmia was present in 68 of 95 pRBD+ participants (71.6%) and 67 of 106 pRBD− participants (63.2%); this difference was not statistically significant (*p* = 0.208). Disease severity was mild to moderate: 169 participants (84.1%) were at Hoehn and Yahr stages 1–2, 29 (14.4%) at stage 3, and 3 (1.5%) at stage 4.

**TABLE 1:** Baseline characteristics of the study cohort (*n* = 201).

Variable	*n* (%)	Median (IQR)	*n*
Age (years)	–	66.0 (10.0)	201
Sex	–	–	
Male	129 (64.2)	–	
Female	72 (35.8)	–	
Age at onset (years)	–	61.0 (12.0)	201
Duration (months)	–	48.0 (70.5)	196
LEDD (mg)	–	479.0 (538.9)	201
pRBD	95 (47.3)	–	
Anosmia	135 (67.2)	–	
pR+A+	68 (33.8)	–	
pR+A−	27 (13.4)	–	
pR−A+	67 (33.3)	–	
pR−A−	39 (19.4)	–	
Onset side	–	–	
Unilateral	158 (78.6)	–	
Bilateral	37 (18.4)	–	
Not applicable	6 (3.0)	–	
Hoehn and Yahr stage		2 (0)	201
Stage 1–2	169 (84.1)		
Stage 3–4	32 (15.9)		
UPSIT	–	14.0 (9.0)	201
MDS-UPDRS total score	–	45.0 (24.0)	199
MDS-UPDRS part III score	–	27.5 (14.0)	200
NMSS	–	23.0 (33.0)	169
MoCA	–	26.0 (4.3)	201
MMSE		28.0 (4.0)	201
PDQ-39 SI	–	11.5 (22.0)	193

A+, anosmia; IQR, interquartile range; LEDD, levodopa-equivalent daily dose; MDS-UPDRS, Movement Disorder Society–Unified Parkinson’s Disease Rating Scale; MMSE, Mini-Mental State Examination; MoCA, Montreal Cognitive Assessment; NA, not applicable; NMSS, Non-Motor Symptoms Scale; PDQ-39 SI, Parkinson’s Disease Questionnaire-39 Summary Index; pRBD and pR+, probable REM sleep behavior disorder; UPSIT, University of Pennsylvania Smell Identification Test.

Data completeness varied by instrument. MoCA, MMSE, and UPSIT data were complete for all participants, whereas MDS-UPDRS total scores were missing for 2 participants (1.0%), part III scores for 1 (0.5%), PDQ-39 SI scores for 8 (4.0%), and NMSS scores for 32 (15.9%); the number included in each analysis is reported in the tables. The proportion of missing PDQ-39 SI and NMSS data did not differ significantly across the four subgroups (*p* = 0.254 and *p* = 0.421, respectively).

Among the 191 participants with interpretable TRODAT SPECT, nigrostriatal denervation was least severe in the pR−A− subgroup and closely similar in the three subgroups carrying at least one marker (Supplementary Table 1).

Age and age at onset were normally distributed and were compared using analysis of variance; disease duration, LEDD, and UPSIT scores were non-normally distributed and were compared using the Kruskal–Wallis test. The four groups (defined by probable RBD status [pR+/pR−] and anosmia status [A+/A−]) differed in age (*p* = 0.001) and age at onset (*p* = 0.029). The pR−A− group was the youngest, whereas the pR−A+ group was the oldest. LEDD also differed across groups (*p* = 0.003), with the lowest dose observed in the pR−A− group (Table 2).

**TABLE 2:** Demographic and clinical characteristics according to probable RBD and anosmia status.

Variable	pR+A+ (*n* = 68)	pR+A− (*n* = 27)	pR−A+ (*n* = 67)	pR−A− (*n* = 39)	*p* ^†^
Age (years)	66.5 (10.0)	67.0 (14.5)	68.0 (7.0)	61.0 (9.5)	0.001*
Sex, *n* (%)	–	–	–	–	0.827
Male	42 (61.8)	17 (63.0)	46 (68.7)	24 (61.5)	–
Female	26 (38.2)	10 (37.0)	21 (31.3)	15 (38.5)	–
Age at onset (years)	60.0 (11.0)	60.0 (16.8)	63.0 (10.0)	58.0 (12.5)	0.029*
Duration (months)	51.0 (62.5)	52.0 (88.3)	47.0 (74.0)	42.0 (46.5)	0.558
LEDD (mg)	639.0 (625.0)	620.0 (439.9)	549.0 (486.8)	314.8 (456.1)	0.003*
Onset side, *n* (%)	–	–	–	–	0.215
Unilateral	53 (77.9)	20 (74.1)	55 (82.1)	30 (77.0)	–
Bilateral	14 (20.6)	4 (14.8)	10 (14.9)	9 (23.0)	–
NA	1 (1.5)	3 (11.1)	2 (3.0)	0 (0.0)	–
Hoehn and Yahr stage					0.175
Stage 1–2	56 (82.4)	23 (85.2)	55 (82.1)	35 (89.7)	
Stage 3–4	12 (17.6)	4 (14.8)	12 (17.9)	4 (10.3)	
UPSIT	12.0 (7.5)	22.0 (4.8)	12.0 (4.0)	22.0 (5.0)	<0.001*

Continuous variables are presented as median (IQR), and categorical variables as *n* (%).

† *p* values were calculated using analysis of variance (age and age at onset), the Kruskal–Wallis test (disease duration, LEDD, and UPSIT), or Chi-squared tests (sex, side of onset, and Hoehn and Yahr stage), as appropriate.

*p < 0.05. pRBD+ participants were considered more likely to correspond to the body-first subtype and pRBD− participants to the brain-first subtype. IQR, interquartile range; LEDD, levodopa-equivalent daily dose; NA, not applicable; pRBD, probable REM sleep behavior disorder; UPSIT, University of Pennsylvania Smell Identification Test. pR+A+, positive for both probable RBD and anosmia; pR+A−, positive for probable RBD and negative for anosmia; pR−A+, negative for probable RBD and positive for anosmia; pR−A−, negative for both probable RBD and anosmia.

In unadjusted comparisons, the pR+A+ and pR+A− groups did not differ in any assessed outcome (Figure 1). MDS-UPDRS total and part III scores differed across the four groups (both *p* = 0.001), with the lowest scores in the pR−A− group. The pR−A− group also had the lowest LEDD (*p* = 0.003). MoCA scores differed across groups (*p* < 0.001) and were lowest in the pR−A+ group. MMSE scores showed the same pattern as MoCA scores, with the lowest scores in the pR−A+ group (Supplementary Figure 1). The pR+A+ group had higher NMSS scores than the pR−A− group (Conover *p* < 0.001) and higher PDQ-39 SI scores than the pR−A+ and pR−A− groups (Conover *p* = 0.001 and *p* < 0.001, respectively). After adjustment for age, sex, and disease duration, pR+A+ and pR+A− remained similar for MDS-UPDRS total score (adjusted mean difference 2.2, 95% CI −8.7 to 13.1; *p* = 0.693), MDS-UPDRS part III (3.4, −2.2 to 9.0; *p* = 0.231), MoCA (0.2, −1.7 to 2.1; *p* = 0.819), NMSS (5.6, −11.4 to 22.6; *p* = 0.516), and PDQ-39 SI (4.8, −4.4 to 13.9; *p* = 0.308).

**FIGURE 1:**
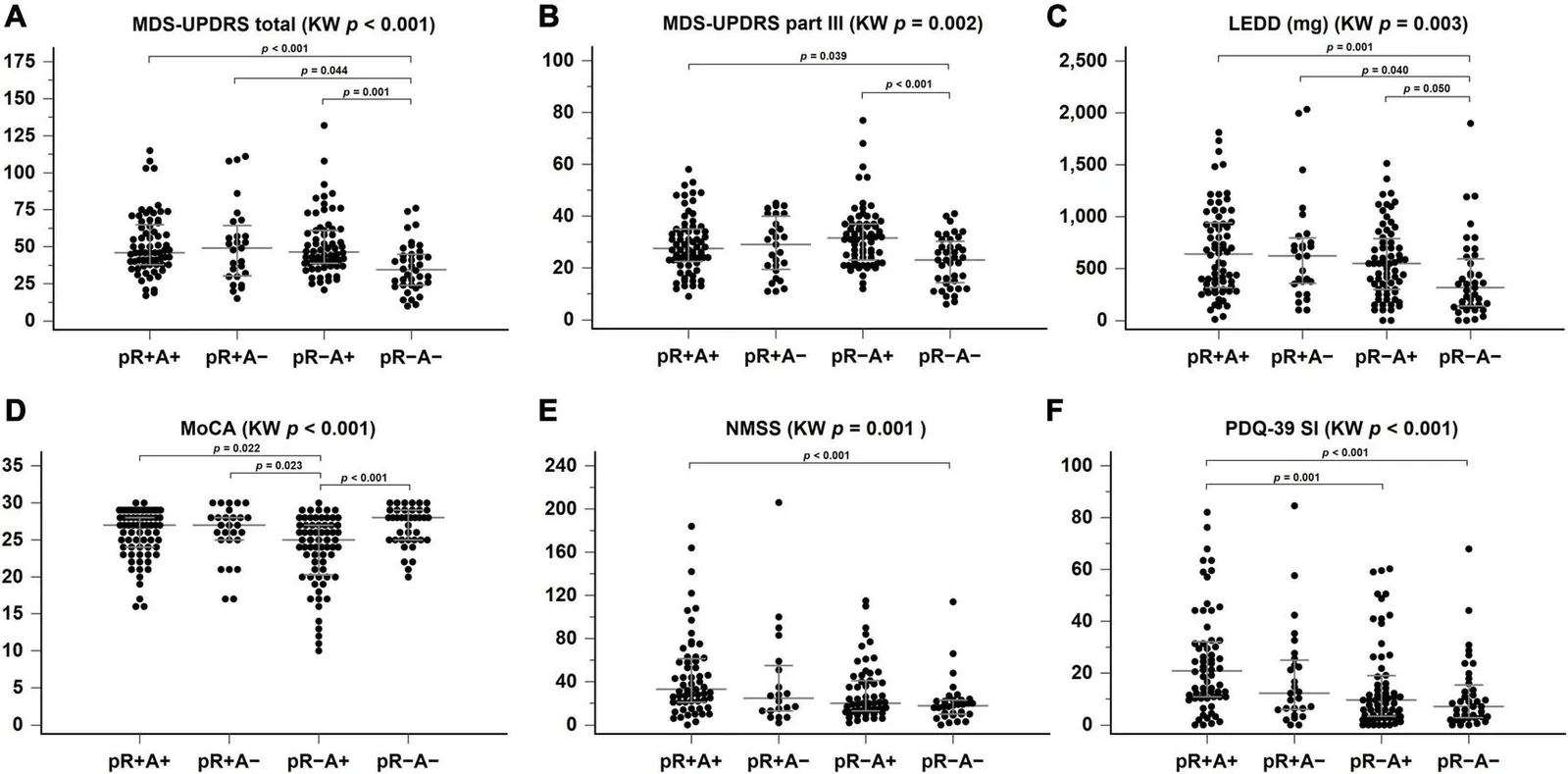
Kruskal–Wallis and Conover tests were used for unadjusted group comparisons. Panels show **(A)** MDS-UPDRS total score, **(B)** MDS-UPDRS part III score, **(C)** LEDD, **(D)** MoCA, **(E)** NMSS, and **(F)** PDQ-39 SI. Pairwise *p* values were obtained using Conover’s test with Bonferroni adjustment. Horizontal lines indicate group medians and error bars the interquartile range. pR+A+, positive for both probable RBD and anosmia; pR+A−, positive for probable RBD and negative for anosmia; pR−A+, negative for probable RBD and positive for anosmia; pR−A−, negative for both probable RBD and anosmia. pRBD+ participants were considered more likely to correspond to the body-first subtype and pRBD− participants to the brain-first subtype. KW, Kruskal–Wallis test; LEDD, levodopa-equivalent daily dose; MDS-UPDRS, Movement Disorder Society–Unified Parkinson’s Disease Rating Scale; MoCA, Montreal Cognitive Assessment; NMSS, Non-Motor Symptoms Scale; PDQ-39 SI, Parkinson’s Disease Questionnaire-39 Summary Index.

The independent main effects of anosmia and pRBD and their interaction were evaluated using factorial linear regression adjusted for age, sex, and disease duration (Table 3; Supplementary Table 2). Anosmia and pRBD were each associated with higher MDS-UPDRS total scores (anosmia: β = 11.9, 95% CI 3.3 to 20.6; *p* = 0.007; pRBD: β = 13.8, 95% CI 3.6 to 24.1; *p* = 0.008). For MDS-UPDRS part III, the anosmia coefficient was significant (β = 9.1, 95% CI 4.3 to 13.8; *p* < 0.001), whereas the pRBD coefficient did not reach statistical significance (β = 5.6, 95% CI 0.0 to 11.2; *p* = 0.051). pRBD was associated with a higher LEDD (β = 218 mg, 95% CI 31 to 405; *p* = 0.022), whereas the anosmia main effect and anosmia-by-pRBD interaction for LEDD were not significant. Anosmia was associated with lower MoCA scores (β = −2.6, 95% CI −4.1 to −1.0; *p* = 0.001), whereas the pRBD main effect was not significant (β = −0.5; 95% CI −2.3 to 1.4; p = 0.628). The anosmia-by-pRBD interaction was significant for MDS-UPDRS part III (β = −8.3, 95% CI −15.2 to −1.4; *p* = 0.019) and MoCA (β = 2.3, 95% CI 0.0 to 4.5; *p* = 0.049), with a non-significant trend for MDS-UPDRS total score (β = −11.9, 95% CI −24.5 to 0.7; *p* = 0.064). The direction of these interaction coefficients indicated attenuation of the individual associations when anosmia and pRBD co-occurred. No main or interaction effects were significant for NMSS or PDQ-39 SI.

**TABLE 3:** Factorial linear regression analysis of the main and interactive effects of anosmia and probable RBD.

Outcome	Anosmia main effect (β, *p*)	pRBD main effect (β, *p*)	Anosmia × pRBD interaction (β, *p*)
MDS-UPDRS total	+11.9, *p* = 0.007*	+13.8, *p* = 0.008*	−11.9, *p* = 0.064
MDS-UPDRS part III	+9.1, *p* < 0.001*	+5.6, *p* = 0.051	−8.3, *p* = 0.019*
LEDD	+107, *p* = 0.183	+218, *p* = 0.022*	−109, *p* = 0.350
MoCA	−2.6, *p* = 0.001*	−0.5, *p* = 0.628	+2.3, *p* = 0.049*
NMSS	+4.2, *p* = 0.588	+15.9, *p* = 0.095	+1.4, *p* = 0.907
PDQ-39 SI	+2.0, *p* = 0.595	+5.6, *p* = 0.197	+4.3, *p* = 0.424

The model was adjusted for age, sex, and disease duration. Regression coefficients are presented with *p* values; 95% confidence intervals for all coefficients are provided in Supplementary Table 2. pRBD+ participants were considered more likely to correspond to the body-first subtype and pRBD− participants to the brain-first subtype. Opposite signs for the main-effect and interaction coefficients indicate attenuation when anosmia and pRBD co-occurred. Anosmia and pRBD had positive coefficients for MDS-UPDRS part III, whereas the anosmia-by-pRBD interaction was negative. For MoCA, the anosmia coefficient was negative and the interaction coefficient was positive. No main or interaction coefficients were significant for NMSS or PDQ-39 SI. β, regression coefficient; CI, confidence interval; A, anosmia; pR, probable RBD; A × pR, anosmia-by-pRBD interaction; LEDD, levodopa-equivalent daily dose; MDS-UPDRS, Movement Disorder Society–Unified Parkinson’s Disease Rating Scale; MoCA, Montreal Cognitive Assessment; NMSS, Non-Motor Symptoms Scale; PDQ-39 SI, Parkinson’s Disease Questionnaire-39 Summary Index.

*p < 0.05.

Multivariable logistic regression was used to identify factors associated with cognitive impairment (MoCA < 26), with adjustment for age, sex, disease duration, and LEDD (Table 4). In the full cohort (*n* = 196; 84 events), anosmia was not significantly associated with cognitive impairment (OR 1.85, 95% CI 0.96–3.56; *p* = 0.066), whereas older age was independently associated with cognitive impairment (OR 1.04 per year, 95% CI 1.00–1.09; *p* = 0.038). pRBD (OR 0.55, 95% CI 0.30–1.00; *p* = 0.051), sex, disease duration, and LEDD were not significant. In stratified analyses, anosmia was associated with cognitive impairment among pRBD− participants (OR 2.53, 95% CI 1.00–6.37; *p* = 0.049), but not among pRBD+ participants (OR 1.65, 95% CI 0.60–4.50; *p* = 0.331).

**TABLE 4:** Multivariable logistic regression analysis of cognitive impairment (MoCA < 26).

Model/predictor	OR (95% CI)	*p*
Whole cohort (n = 196, events = 84)		
Anosmia	1.85 (0.96–3.56)	0.066
pRBD	0.55 (0.30–1.00)	0.051
Age (per year)	1.04 (1.00–1.09)	0.038*
Sex (male)	0.74 (0.39–1.39)	0.349
Disease duration	1.00 (0.99–1.00)	0.415
LEDD (per 100 mg)	1.06 (0.98–1.15)	0.143
Stratified: pRBD+ (body-first) (n = 94, events = 35)		
Anosmia	1.65 (0.60–4.50)	0.331
Stratified: pRBD− (brain-first) (n = 102, events = 49)		
Anosmia	2.53 (1.00–6.37)	0.049*

The model was adjusted for age, sex, disease duration, and LEDD. pRBD+ participants were considered more likely to correspond to the body-first subtype and pRBD− participants to the brain-first subtype. Cognitive impairment was defined as MoCA < 26. The full-cohort model included anosmia, pRBD, and all covariates; stratified models were fitted separately within the pRBD+ and pRBD− subgroups. CI, confidence interval; LEDD, levodopa-equivalent daily dose; MoCA, Montreal Cognitive Assessment; OR, odds ratio; pRBD, probable REM sleep behavior disorder.

*p < 0.05.

Overall, the prevalence of anosmia did not differ significantly between pRBD+ and pRBD− participants. Within the pRBD+ subgroup, anosmia was not associated with worse motor, cognitive, non-motor, or quality-of-life outcomes, and its interaction with pRBD was sub-additive for motor severity. By contrast, anosmia was associated with cognitive impairment within the pRBD− subgroup.

## Discussion

Our study had four principal findings. First, 47.3% of participants were pRBD+, 67.2% had anosmia, 33.8% had both, and 33.3% had isolated anosmia (pR−A+); although anosmia was more frequent in the pRBD+ than in the pRBD− subgroup, the difference was not statistically significant (71.6% vs. 63.2%). Second, after adjustment for age, sex, and disease duration, the pR+A+ and pR+A− groups did not differ in motor severity, cognition, non-motor symptoms, or quality of life. The negative anosmia-by-pRBD interaction for MDS-UPDRS part III further indicated a sub-additive, rather than cumulative, association with motor severity. Third, pRBD+ participants generally had a greater motor and non-motor disease burden than pRBD− participants. Finally, the association between anosmia and cognition was concentrated in the pRBD− subgroup: pR−A+ participants had the lowest MoCA scores, and anosmia was associated with cognitive impairment in pRBD− but not pRBD+ participants.

Taken together, these findings suggest that anosmia and pRBD do not behave as two independent clinical hits whose effects simply add to disease severity. The absence of additional motor burden in pR+A+ relative to pR+A− is compatible with these groups sharing a body-first trajectory in which olfactory involvement occurs within the same broader disease process. Conversely, the cognitive vulnerability observed in pR−A+ participants raises the possibility that isolated anosmia identifies a distinct pRBD− phenotype involving olfactory-limbic networks.

The prevalence of pRBD in our cohort (47.3%) slightly exceeded the previously reported estimates (18.8%–47.2%) (Rolinski et al., 2014; Liu et al., 2021; Chen et al., 2022; Solla et al., 2023). This variability may reflect differences in screening thresholds, definitions of disease duration, and whether RBD status was assessed at a single visit or longitudinally. In the Oxford Discovery Cohort, the prevalence of pRBD was 47.2% using an RBDSQ cutoff of 5, but 37.1% using a cutoff of 6 (Rolinski et al., 2014). A reanalysis of the same cohort requiring positive screening at repeated visits (cutoff 6) reported a prevalence of 33.3% (Liu et al., 2021). A de novo cohort screened with a cutoff of 5 reported pRBD in 37.6% of participants (Chen et al., 2022), whereas a drug-naïve cohort using a higher threshold reported possible RBD in 18.8% (Solla et al., 2023). An estimate restricted to premotor RBD was 20.3% using the REM Sleep Behavior Disorder Single-Question Screen (Velucci et al., 2025). Differences in disease stage may partly account for this variation. The Oxford Discovery Cohort measured duration from diagnosis (1.5 years) rather than onset, to limit recall bias, whereas we measured from motor symptom onset. Its baseline figures (2.9 years from onset, 1.2 years from diagnosis) imply an onset-to-diagnosis interval of about 1.7 years; applying this interval places that cohort at roughly 3.2 years from onset (Rolinski et al., 2014). On this onset-based scale, the comparison cohorts averaged approximately 38 months versus a median of 48 months in ours. Our participants were therefore modestly, rather than substantially, further into the disease course, suggesting that disease stage alone is unlikely to fully explain the difference. Other contributors include recruitment from a single tertiary movement-disorder clinic rather than population-based or multicenter research cohorts, single-visit rather than repeated ascertainment, and the performance of the locally validated questionnaire. Concurrent olfactory impairment and pRBD have been reported in approximately 15.2%–32.7% of individuals with early PD; the prevalence of 33.8% in our cohort slightly exceeded the upper end of this range (Kang et al., 2016; Chen et al., 2022). Anosmia was numerically more frequent in pRBD+ than pRBD− participants, consistent with the direction predicted for body-first PD (Horsager et al., 2022), but the difference was not statistically significant. This finding, together with questionnaire-based rather than polysomnographic classification of RBD, precludes firm conclusions about subtype-specific prevalence.

The greater overall disease burden observed in pRBD+ participants is consistent with studies reporting more severe non-motor manifestations in pRBD+ PD (Rolinski et al., 2014; Solla et al., 2023; Xu et al., 2024). Within the body-first framework, this pattern has been attributed to earlier lower-brainstem involvement and, with disease progression, a more widespread α-synuclein burden (Postuma and Berg, 2016; Horsager et al., 2022). The higher motor burden and LEDD in the pRBD+ group are also broadly consistent with previous subgroup comparisons (Kang et al., 2016; Chen et al., 2022; Solla et al., 2023; Xu et al., 2024) and with proposed multisynaptic propagation toward the substantia nigra (Borghammer and Van Den Berge, 2019; Borghammer et al., 2021).

The differential association between anosmia and cognition according to pRBD status requires cautious interpretation. Our findings are consistent with a machine-learning SuStaIn analysis that identified one trajectory characterized by early hyposmia and cognitive impairment and another characterized by early pRBD, hyposmia, and autonomic dysfunction (Zhou et al., 2023). Our findings also align with broader evidence linking olfactory impairment to cognitive decline, smaller hippocampal, entorhinal, fusiform, and middle temporal volumes (Dintica et al., 2019), and frontal structural abnormalities (Okumura et al., 2024). In contrast, a previous study modeled hyposmia and pRBD as a cumulative prodromal burden, grouping participants by the number of factors present (neither, one, or both) and reporting progressively poorer cognition with increasing burden (Kang et al., 2016). However, that ordinal design merged the two single-factor groups (hyposmia alone and pRBD alone) into one category and assumed a monotonic dose–response, thereby precluding estimation of each factor’s independent contribution or of their interaction. Using a factorial design that modeled the main effects of anosmia and pRBD and their interaction, we found the two did not behave as simple additive hits. Disaggregating the two factors therefore reveals a pattern that departs from a monotonic burden model, with the cognitive relevance of anosmia emerging specifically in the absence of pRBD. Using operational definitions comparable to ours in a larger, longitudinal cohort, a study found that faster cognitive decline was confined to pRBD+ groups, whereas isolated olfactory impairment (pRBD− individuals meeting the same UPSIT anosmia threshold) did not accelerate cognitive decline (Chen et al., 2022). This apparent contrast with our results reflects two differences: our analysis was cross-sectional, modeling prevalent cognitive impairment (MoCA < 26) rather than the rate of decline, and it explicitly tested the anosmia-by-pRBD interaction and pRBD− stratified effects, which the four-group comparison did not. The cognitive relevance of anosmia within the pRBD− subgroup should therefore be regarded as a cross-sectional, hypothesis-generating finding awaiting longitudinal confirmation. Another longitudinal study reported more frequent hyposmia and a greater cognitive burden in body-first PD, although hyposmia was self-reported and cognition was assessed with a single UPDRS part I item (Velucci et al., 2025). Moreover, the association between isolated anosmia and cognitive impairment in our cohort differs from a prevailing formulation in which hyposmia reflects total cerebral Lewy body burden and should therefore be more frequent in body-first PD (Borghammer et al., 2022).

Clinically, concurrent assessment of olfaction and probable RBD may provide more informative phenotyping than either marker alone. Anosmia was present in 67.2% of our cohort and was therefore a usual finding with limited discriminative value; its significance depended on pRBD status. Under a dual-hit framework, in which olfactory and enteric routes are affected separately, the two markers might be expected to carry additive burden; no such increment was seen once pRBD was present, which is more consistent with the revised single-hit framework. Their burdens therefore cannot simply be added: what matters clinically is which combination an individual has. In PD, cognitive risk is usually signaled by pRBD. Our cross-sectional data suggest that anosmia may carry that signal when pRBD is absent: cognition therefore warrants closer attention in pR−A+ individuals, whereas pRBD+ individuals warrant closer surveillance of overall motor and non-motor burden. These inferences remain indirect: pRBD marks the presence rather than the timing of RBD and therefore identifies body-first PD only imperfectly, and because olfactory performance may not correspond directly to olfactory bulb pathology, the anatomical origin cannot be established here.

Several limitations should be considered. First, pRBD was classified using the RBDSQ rather than gold-standard video-polysomnography, which may have introduced misclassification. Nevertheless, the RBDSQ is a practical first-step screening instrument for large cohorts and outpatient settings (Bloem et al., 2021; Liu et al., 2021). At a cutoff of 5, the original validation reported a sensitivity of 0.96 and a specificity of 0.56 (Stiasny-Kolster et al., 2007), whereas the validated Taiwan version reported a sensitivity of 0.67, a specificity of 0.68, and an area under the curve of 0.75 (Fang et al., 2025). In individuals with PD, a cutoff of 6 yielded a sensitivity of 0.84, a specificity of 0.96, and fair internal consistency (Cronbach’s α = 0.73) (Nomura et al., 2011). The Oxford Discovery cohort suggested a cutoff of 6, since a confirmed PD diagnosis should produce a positive response to the RBDSQ’s final item (Liu et al., 2021). Thus, the RBDSQ combined with participant and informant interviews is appropriate for identifying pRBD in PD but does not substitute for polysomnographic confirmation, the major limitation of this study. Second, the cohort did not include de novo or drug-naïve individuals, and the premotor onset of pRBD was not ascertained; pRBD+ and pRBD− participants were treated as approximate proxies for the body-first and brain-first subtypes rather than definitive classifications. Third, as a single-center study with a modest sample size, it has lower statistical power and generalizability than multicenter PPMI-based studies (Chen et al., 2022; Solla et al., 2023). The subgroup analyses further reduced statistical power. Fourth, the cross-sectional design precluded assessment of temporal sequence or progression, unlike longitudinal subtype studies (Chen et al., 2022; Velucci et al., 2025). Fifth, some instruments had incomplete data – particularly the NMSS (15.9% missing) – and were analyzed as complete cases without imputation. Sixth, the broad ranges of disease duration and LEDD indicate clinical heterogeneity within the cohort. Seventh, dopamine transporter imaging was interpreted with an ordinal visual scale rather than quantitative striatal binding ratios. Because almost half of the participants were graded severe in both putamina, the scale has insufficient dynamic range to assess the left–right symmetry of denervation. Quantitative dopamine transporter studies have reported more symmetric striatal denervation in PD with premotor RBD (Woo et al., 2024), although this has not been confirmed in another cohort with polysomnography-confirmed RBD (Lövdal et al., 2024); testing this prediction would require quantitative binding ratios. Future studies should incorporate longitudinal follow-up, polysomnographic confirmation, imaging or other subtype biomarkers, and monorhinal olfactory testing to assess clinically silent unilateral olfactory loss and asymmetric propagation.

In conclusion, the clinical association of anosmia differed according to pRBD status. In pRBD+ participants, anosmia was not associated with greater motor severity; in pRBD− participants, it was instead linked to cognitive impairment. Concurrent assessment of olfaction and pRBD may therefore improve clinical phenotyping.

## Data Availability

The raw data supporting the conclusions of this article will be made available by the authors, without undue reservation.
