# Supplementary material for: Olfactory impairment and probable REM sleep behavior disorder in Parkinson’s disease: clinical relevance to the single- and dual-hit hypotheses
Source: Front Aging Neurosci. 2026 Sep 22;18:1778012. doi: 10.3389/fnagi.2026.1778012 (PMC13638651; doi:10.3389/fnagi.2026.1778012)
Supplement: Supplementary file 1 [file Data_Sheet_1.pdf]

Supplementary Figures and Tables.

Table 1. TRODAT imaging visual classification according to probable RBD and anosmia status

	pR+A+ (n = 63)	pR+A− (n = 27)	pR−A+ (n = 63)	pR−A− (n = 38)	*p*
Striatal grade (0–6)	4.5 (1.1)	4.5 (1.3)	4.5 (1.3)	3.5 (1.5)	0.010*
Overall visual scale (0–5)	3.5 (1.0)	3.5 (1.5)	3.0 (1.5)	2.5 (1.5)	0.082
Worse-side putamen (0–6)	6.0 (0.0)	6.0 (0.0)	6.0 (0.0)	6.0 (1.0)	0.113
Worse-side caudate (0–6)	4.0 (2.0)	4.0 (2.0)	4.0 (2.0)	3.0 (2.0)	0.146

Interpretable TRODAT SPECT was available for 191 of the 201 participants. The visual grades were coded ordinally (0 = normal to 6 = severe), while the overall visual scale used a 0–5 range. Values are presented as median (IQR). The Kruskal–Wallis test compared the four subgroups; *$p < 0.05$.

Figure 1. MMSE and MoCA according to probable RBD and anosmia status

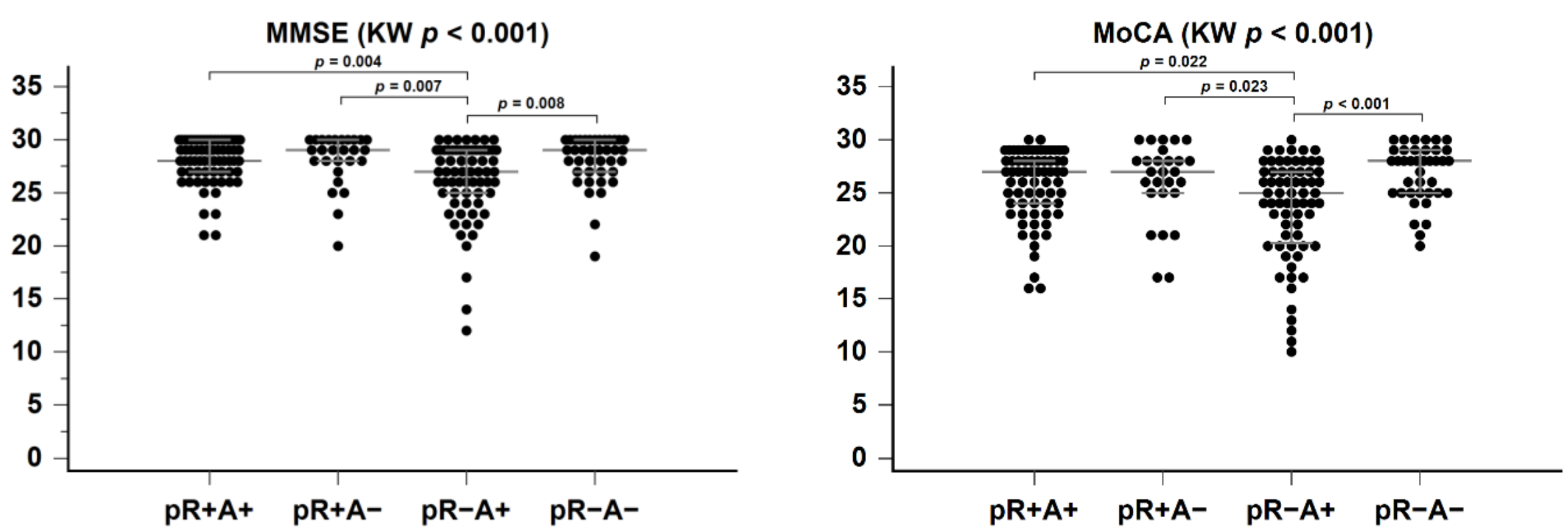

MMSE data were complete for all 201 participants. Scores (median [IQR]) were 28.0 (3.0) in the pR+A+ subgroup, 29.0 (2.0) in pR+A−, 27.0 (4.0) in pR−A+, and 29.0 (3.0) in pR−A−. The overall difference was tested with the Kruskal–Wallis test; pairwise post-hoc comparisons used Conover's all-pairs test with Bonferroni correction (two-sided). The result was in the same direction as that for the MoCA.

Table 2. Factorial linear regression analysis of the main and interactive effects of anosmia and probable RBD, with 95% confidence intervals

Outcome	Anosmia main effect (*β*, 95% CI, *p*)	pRBD main effect (*β*, 95% CI, *p*)	Anosmia × pRBD interaction (*β*, 95% CI, *p*)
MDS-UPDRS total	+11.9 (3.3 to 20.6), $p = 0.007$*	+13.8 (3.6 to 24.1), $p = 0.008$*	−11.9 (−24.5 to 0.7), $p = 0.064$
MDS-UPDRS part III	+9.1 (4.3 to 13.8), $p < 0.001$*	+5.6 (0.0 to 11.2), $p = 0.051$	−8.3 (−15.2 to −1.4), $p = 0.019$*
LEDD	+107 (−51 to 264), $p = 0.183$	+218 (31 to 405), $p = 0.022$*	−109 (−339 to 121), $p = 0.350$
MoCA	−2.6 (−4.1 to −1.0), $p = 0.001$*	−0.5 (−2.3 to 1.4), $p = 0.628$	+2.3 (0.0 to 4.5), $p = 0.049$*
NMSS	+4.2 (−11.2 to 19.6), $p = 0.588$	+15.9 (−2.8 to 34.7), $p = 0.095$	+1.4 (−21.0 to 24.0), $p = 0.907$
PDQ-39 SI	+2.0 (−5.3 to 9.3), $p = 0.595$	+5.6 (−2.9 to 14.2), $p = 0.197$	+4.3 (−6.3 to 14.9), $p = 0.424$

The model was adjusted for age, sex, and disease duration. This table presents the same analysis as Table 3 of the main text, with 95% confidence intervals shown for all coefficients.

Supplementary References

Dalrymple-Alford, J. C., MacAskill, M. R., Nakas, C. T., Livingston, L., Graham, C., Crucian, G. P., et al. (2010). The MoCA: well-suited screen for cognitive impairment in Parkinson disease. Neurology 75, 1717–1725. doi: 10.1212/WNL.0b013e3181fc29c9

Hoops, S., Nazem, S., Siderowf, A. D., Duda, J. E., Xie, S. X., Stern, M. B., et al. (2009). Validity of the MoCA and MMSE in the detection of MCI and dementia in Parkinson disease. Neurology 73, 1738–1745. doi: 10.1212/WNL.0b013e3181c34b47

Huang, W. S., Lee, M. S., Lin, J. C., Chen, C. Y., Yang, Y. W., Lin, S. Z., et al. (2004). Usefulness of brain 99mTc-TRODAT-1 SPET for the evaluation of Parkinson's disease. Eur. J. Nucl. Med. Mol. Imaging 31, 155–161. doi: 10.1007/s00259-003-1331-x
